# Texture analysis of MR images to identify the differentiated degree in hepatocellular carcinoma: a retrospective study

**DOI:** 10.1186/s12885-020-07094-8

**Published:** 2020-06-30

**Authors:** Mengmeng Feng, Mengchao Zhang, Yuanqing Liu, Nan Jiang, Qian Meng, Jia Wang, Ziyun Yao, Wenjuan Gan, Hui Dai

**Affiliations:** 1 Department of Radiology, the First Affiliated Hospital of Soochow University, Suzhou city, Jiangsu province 215000 P.R. China; 2 Department of Radiology, the China-Japan Union Hospital of Jilin University, Changchun city, Jilin province 130033 P.R. China; 3 Department of Hepatobiliary Surgery Department, the First Affiliated Hospital of Soochow University, Suzhou city, Jiangsu province 215000 P.R. China; 4 Department of Pathology Department, the First Affiliated Hospital of Soochow University, Suzhou city, Jiangsu province 215000 P.R. China; 5 Institute of Medical Imaging, Soochow University, Suzhou city, Jiangsu province 215000 P.R. China

**Keywords:** Hepatocellular carcinoma, Differentiated degree, Texture feature

## Abstract

**Background:**

To explore the clinical value of texture analysis of MR images (multiphase Gd-EOB-DTPA-enhanced MRI and T2 weighted imaging (T2WI) to identify the differentiated degree of hepatocellular carcinoma (HCC).

**Method:**

One hundred four participants were enrolled in this retrospective study. Each participant performed preoperative Gd-EOB-DTPA-enhanced MR scanning. Texture features were analyzed by MaZda, and B11 program was used for data analysis and classification. The diagnosis efficiencies of texture features and conventional imaging features in identifying the differentiated degree of HCC were assessed by receiver operating characteristic analysis. The relationship between texture features and differentiated degree of HCC was evaluated by Spearman’s correlation coefficient.

**Results:**

The grey-level co-occurrence matrix -based texture features were most frequently extracted and the nonlinear discriminant analysis was excellent with the misclassification rate ranging from 3.33 to 14.93%. The area under the curve (AUC) of the combined texture features between poorly- and well-differentiated HCC, poorly- and moderately-differentiated HCC, moderately- and well-differentiated HCC was 0.812, 0.879 and 0.808 respectively, while the AUC of tumor size was 0.649, 0.660 and 0.517 respectively. The tumor size was significantly different between poorly- and moderately-HCC (*p* = 0.014). The COMBINE AUC values were not increased with tumor size combined.

**Conclusions:**

Texture analysis of Gd-EOB-DTPA-enhanced MRI and T2WI was valuable and might be a promising method in identifying the differentiated degree of HCC. The poorly-differentiated HCC was more heterogeneous than well- and moderately-differentiated HCC.

## Background

Hepatocellular carcinoma (HCC) is a malignant tumor evolved from the hepatocyte and is the second most common cause of cancer death worldwide. HCC account for a larger proportion of tumor particularly in developing countries [1]. The high prevalence of hepatitis virus B is the most common reason leading to HCC in the developing countries, while the alcohol and hepatitis C virus is more frequent in developed countries. Although there are many treatments of HCC including surgery, radiofrequency ablation and transcatheter arterial chemoembolization, the mortality of HCC is still high due to the recurrence [2].

There were many reports suggested that the size of tumor, number of lesion, vascular invasion, status of tumor capsule and liver function status can affect the prognosis and the choices of therapy of HCC [3–6]. Nevertheless, the most important factor was the differentiated grade, which was supposed to an independent factor affecting recurrence of HCC [7]. According to the differentiated degree of tumor cells, HCC were grouped into poorly-differentiated HCC, moderately-differentiated HCC and well-differentiated HCC. According to the reports, the overall survival rate of the patients with moderately-differentiated and well-differentiated HCC was higher than that of the patients with poorly-differentiated HCC, while the recurrence rate was lower [8, 9].

As we known, a precise pre-surgical evaluation of differentiated degree of HCC might affect the individual treatment schedule [10]. Currently, aspiration biopsy was the most common method to get the information of histopathology before surgery. However, it was criticized by many researchers due to its invasiveness and the probability of seeding metastasis [11, 12]. Recently, many studies suggested the image characteristics of tumor might predict the differential degree of the HCC. For example, there were some reports found that the low density/intensity of HCC on the portal phase of CT and hepatobiliary phase of Gd-EOB-DTPA-enhanced MRI might help to identify the differentiated degree of HCC [13, 14].

Texture analysis was an established technique, which was beneficial to diagnoses, by extracting a large amount of texture information from medical images [15]. It was used in identifying the differentiated degree and characteristics of tumor, and evaluating the therapeutic effect, etc. [16–18]. However, the texture analysis has not been used in identifying the differentiated degree of HCC yet. Thus, our aim of the present study is to evaluate the accuracy of the texture analysis of MR images in discriminating the differentiated degree of HCC, and to compare the diagnostic efficiencies of conventional imaging features and texture features.

## Methods

### Patients

The present study received ethical approval from the Medical Ethics Review Committee of our institution and the relevant informed consent form was obtained in accordance with the Declaration of Helsinki. One hundred four participants were enrolled from 2015 to 2019, according to the following criteria:1) pathologically proved as HCC after hepatectomy; 2) inpatients who have comprehensive clinic materials; 3) performed preoperative Gd-EOB-DTPA-enhanced MRI. The clinic data of the 104 participants were recorded in the Table 1, containing age, gender, alpha fetoprotein (AFP), alamine aminotransferase (ALT), aspartate transaminase (AST), ALT\AST, total bilirubin (TBIL), direct bilirubin and indirect bilirubin.

**Table 1:** The clinical data of each subtype group and inter-group differences

Parameter	C	A	B	*P* value (A verse C)	*P* value (B verse C)	*P* value (A verse B)
Age	56.647 ± 9.652	59.875 ± 12.522	58.232 ± 10.831	0.356	0.304	0.937
Gender (female\male)	6\31	4\20	7\36	0.963	0.994	0.967
AFP (positive\negative)	32\5	11\13	30\13	0.001	0.074	0.054
ALT	64.705 ± 65.452	116.47 ± 105.389	68.047 ± 77.362	0.006	0.893	0.008
AST	46.430 ± 55.668	72.988 ± 109.165	60.842 ± 123.950	0.063	0.919	0.082
ALT\AST	1.55 ± 0.79	2.070 ± 10.96	1.504 ± 0.752	0.092	0.728	0.044
TBIL	25.695 ± 21.309	25.054 ± 14.022	53.532 ± 95.849	0.488	0.271	0.744
Direct bilirubin	13.483 ± 12.930	12.595 ± 9.154	28.574 ± 50.347	0.626	0.369	0.759
Indirect bilirubin	12.208 ± 9.239	12.463 ± 6.494	27.284 ± 53.889	0.425	0.191	0.724
GPC-3(positive\negative)	17\3	6\5	17\3	0.095	1	0.095

Note: A: well-differentiated HCC, B: moderately-differentiated HCC, C: poorly-differentiated HCC, *AFP* alpha fetoprotein, *ALT* alamine aminotransferase, *AST* aspartate transaminase, *TBIL* total bilirubin, GPC-3: glypican-3

Exclusion criteria included:1) participants have been treated (transplantation, resection, ablation or embolization) before MR examination; 2) clinical data (AFP, ALT, AST, TBIL, direct bilirubin and indirect bilirubin) or pathological results were incomplete; 3) the lesions were not clearly displayed on the images due to the artifact.

### MRI examination

All MRI examinations were performed using 3.0 T MRI machine (Siemens Magnetom Verio 3.0 T; Siemens Magnetom Skyra 3.0 T; GE Signa HDxt 3.0 T) with a dedicated phased-array body coil. A standard abdominal MRI protocol containing following sequences were acquired: 1) Axial T2-weighted: TR = 3260 ms, TE = 105 ms, slice thickness 7 mm, intersection gap 1.4 mm, field of view (FOV) 210 mm × 380 mm; 2) In-phase and out-of-phase axial T1-weighted imaging: TR = 4.16 ms, TE = 2.58 ms (in-phase), TE = 1.35 ms (out-phase), slice thickness 5 mm, intersection gap 1 mm, FOV 210 mm × 380 mm; 3) Diffusion-weighted imaging (DWI, b = 50, 800 s/mm^2^) performed with a free-breathing single-shot echo-planar technique, TR 5300 ms, TE 57 ms, slice thickness 7 mm, intersection gap 1.4 mm, FOV 210 mm × 380 mm; corresponding ADC maps were calculated automatically by a built-in software; and 4) Contrast enhanced MRI, a three-dimensional (3D) gradient echo sequence with volumetric interpolated breath-hold examination (VIBE): TR 4.18 ms, TE 1.93 ms, slice thickness 3 mm without intersection gap, FOV 210 mm × 380 mm. Gd-EOB-DTPA (Primovist, Bayer Healthcare, Berlin, Germany) was used by 0.2 ml/kg with an injection rate of 2 ml/sec. Hepatic arterial phase (AP), portal venous phase (PVP), equilibrium phase (EP) and hepatobiliary phase (HBP) images were obtained.

### Image analysis

The MRI images were reviewed in the picture archiving and communication system (PACS). Experienced radiologists, who were blinded to the pathological results, evaluated the MRI imaging features of the HCC. The imaging features of MRI (arterial enhancement, capsule appearance, the intensity of HBP, the margin and diameter of the tumor, intralesional fat, intratumoral vessel and etc.) were selected referring to the Liver Imaging-Reporting and Data System (LI-RADS 2017) (https://www.acr.org/Clinical-Resources/Reporting-and-Data-Systems/LI-RADS) [19].

### Texture analyses and features selection

MaZda software (version 4.6, quantitative texture analysis software, available from http://www.eletel.p.lodz.pl/mazda/) was used for texture analysis. All images were transformed into Bitmap (BMP) format considering for the application compatibility of MaZda. An experienced radiologist manually portrayed the region of interest (ROI) of the lesion on the slice which contained the maximum proportion of tumor. One hundred four ROIs (one ROI for each patient) on HBP images were analyzed firstly. Subsequently, the ROIs were copied onto T2, AP and EP images. Then, texture features were extracted and analyzed. The texture features could be grouped into grey-level histogram, the grey-level co-occurrence matrix (GLCOM), the grey-level run-length matrix (GLRLM) and wavelet transform. A grey-level histogram indicated how many pixels of an image shared the same grey level. GLCOM was a statistical method of examining image texture, considering the spatial relationship, by calculating how often pairs of pixel with specific values, which could not provide information about shape. The GLRLM gave the size of homogeneous runs for each grey level. Wavelet transforms were a mathematical means for performing signal analysis when signal frequency varied over time. Wavelet transform coefficients could be computed. More detailed texture features were listed in Table 2. Feature selection algorithms included Fisher coefficient, mutual information [MI], and classification error probability combined with average correlation coefficients [POE + ACC]. Ten texture features were extracted by each of these algorithms. In order to enhance the discriminability, these three methods were combined, called “FPM”, by which 30 texture features were extracted in total.

**Table 2:** List of texture features extracted by MaZda software

Main features	More detailed features
Grey-level histogram	Mean, variance, skewness, kurtosis, percentiles (1, 10, 50, 90, 99%)
Grey-level co-occurrence matrix (GLCOM)	Angular second moment, contrast, correlation, entropy, sum entropy, sum of squares, sum average, sum variance, inverse difference moment, difference entropy, difference variance (for four directions and five interpixel distances (offsets; *n* = 1–5))
Grey-level run-length matrix (GLRLM)	Run-length non-uniformity, grey-level non-uniformity, long run emphasis, short run emphasis, fraction of image in runs (for four angles)
Wavelet transform	Energies of wavelet transform coefficients in sub-bands LL, LH, HL, HH

### Histopathological analysis

Histopathological evaluation was available after hepatectomy for the lesions. The specimens were routinely prepared with 4% formaldehyde. The specimens were evaluated by two experienced pathologists who were blind to MRI information. The eight slices of each lesion were analyzed and evaluated with slices ranging from 0.3 cm to 2.0 cm depending on the size of the lesion. The Edmondson-Steiner grade was used to categorize all the specimens. According to the differentiation degree of tumor cells, HCC were categorized into grades I to IV. Edmonson grade I and part of grade II was corresponding with well-differentiated HCC, Edmonson grade II and part of grade III was corresponding with moderately-differentiated HCC, grade III and part of grade IV was poorly-differentiated HCC, and grade IV was undifferentiated HCC. The specimens were stained with Glypican-3 (GPC-3) antibodies. The results of immunohistochemical staining were considered positive if more than 10% of the tumor cells showed cytoplasmic staining, otherwise the results were considered negative.

### Statistical analysis and misclassification rate

The statistical analysis was performed using Statistical Product and Service Software (SPSS ver. 20.0, Chicago, IL). In present study, the group differences of continuous variables in abnormal distribution, such as age, ALT, AST, ALT\AST and texture features, were analyzed by Mann-Whitney U test. The difference of texture features between poorly-, moderately- and well-differentiated HCC were analyzed by Kruskal-Wallis H test. The group differences of categorical variables were analyzed by Pearson Test when the sample size was over 40 and the minimal expected frequency was over 5. Otherwise, the correction formula of chi-squared test would be chosen. And the R × C table was used when the dependent variable was over 2. In order to evaluate the diagnostic accuracy of texture features derived from T2, HBP, AP, and EP, the receiver operating characteristic (ROC) analysis was performed and the area under the curve (AUC) was calculated by MedCalc (MedCalc statistical software, ver.15.8). The correlation between texture features and differentiated degree of HCC was analyzed by Spearman’s correlation coefficient. A p value less than 0.05 was considered statistically significant. And Bonferroni correction was used to adjust p values in multiple comparisons.

The B11, a module of MaZda (version 4.6), provided four analyzing ways - principal component analysis (PCA), linear discriminant analysis (LDA), nonlinear discriminant analysis (NDA) and raw data analysis (RDA), to classify and analyze the texture features. The B11 implemented 1-NN classifier for non-linear supervised classification [20]. The misclassification rate was defined as total false samples divided by the total samples and the ratio indicated that the estimated group was different from the observed group. According to the misclassification rate, the classification results were separate into four levels: excellent (misclassification rates ≤10%), good (10% < misclassification rates ≤20%), moderate (20% < misclassification rates ≤30%), fair (30% < misclassification rates ≤40%), and poor (misclassification rates > 40%) [21].

## Results

### Clinical data

There were 37 patients with poorly-differentiated HCC, 43 with moderately-differentiated HCC, and 24 with well-differentiated HCC in present study. As showed in Table 1, there were no significant differences for age and gender among the groups (*p* > 0.05). There were significant differences for AFP and ALT value between the poor- and well-differentiated HCC (*p* *=* 0.001, 0.006, respectively). The ALT was statistically different between well- and moderately-differentiated HCC (*p* = 0.008). Fifty-one participants were with GPC-3, among which, 20 were with poorly-differentiated HCC, 20 with moderately and 11 with well-differentiated HCC. There was no significant difference of GPC-3 expression among poorly-, well- and moderately-differentiated HCC, as Table 1 showed (*p* > 0.05).

### MRI feature evaluation

**Fig. 1:**
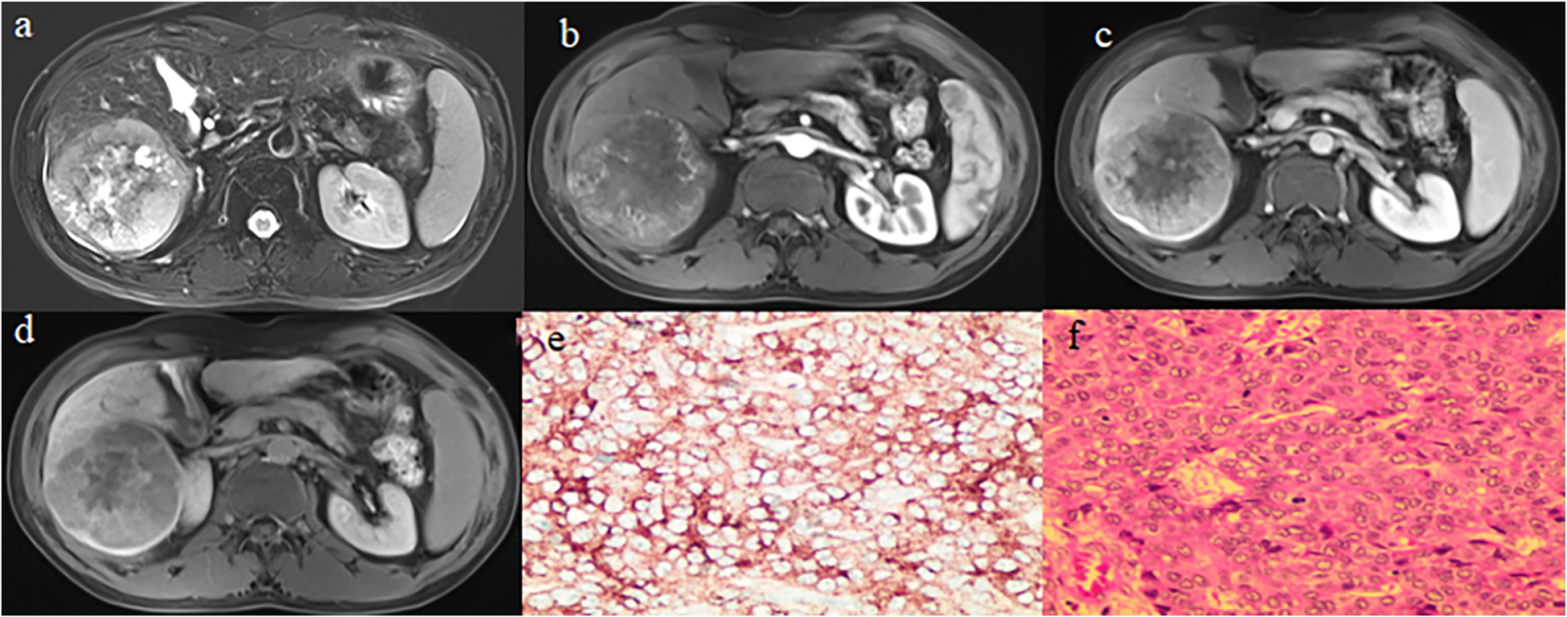
A patient claimed epigastric discomfort and with a history of hepatitis B for several years. As showed in T2WI (**a**), the tumor located in right lobe of liver. T2WI (a) showed heterogeneous signal of the tumor and the complete capsule of the tumor. AP (**b**) images showed the enhancement in the margin of tumor. EP (**c**) images demonstrated the heterogeneous enhancement and non-enhancing center area of the tumor. The tumor showed heterogeneous hypointensity with comparative lower intensity in the center of the tumor on HBP images (**d**). GPC-3 was positive on immunohistochemical examination (×200) (**e**). The pathological result of hematoxylin and eosin staining of tumor section was poorly-differentiated HCC (× 200) (**f)**

The MRI imaging features of l04 patients were demonstrated in Table 3. As the table showed, the tumor size was statistically different between poorly- and moderately-HCC (*p* = 0.014). However, no statistical differences were found in the margin and the capsule status of the tumor, liver cirrhosis, the HBP hypointensity, intratumoral vessel, intralesional fat, rim-enhancement AP and lymphadenectasis, among poorly-, moderately- and well-differentiated HCC. A typical case of poorly-differentiated HCC was showed in Fig. 1.

**Table 3:** MRI features of each subtype group and inter-group differences

Variables	C	A	B	*P* value (A verse C)	*P* value (B verse C)	*P* value (A verse B)
Tumor size	7.16 ± 7.55	4.54 ± 3.29	4.35 ± 3.13	0.051	0.014	0.968
Signal (Homogeneous\Heterogeneous)	12\25	14\10	18\25	0.46	0.524	0.196
Margin (Smooth\Coarse)	17\20	18\6	26\17	0.25	0.19	0.23
Capsule (Complete\Incomplete\None)	17\5\15	12\1\11	22\3\18	0.449	0.615	0.871
Liver cirrhosis (Yes\No)	20\17	12\12	23\20	0.962	0.960	0.784
HBP hypointensity (Yes\No)	7\30	7\17	8\35	0.536	0.971	0.32
Intratumoral vessel (Yes\No)	15\22	7\17	18\25	0.366	0.905	0.303
Intralesional fat (Yes\No)	1\36	3\21	6\37	0.327	0.168	0.867
Rim-enhancement AP (Yes\No)	22\15	12\12	15\28	0.467	0.028	0.226
Lymphadenectasis (Yes\No)	5\32	1\23	1\42	0.449	0.142	1.0

Note: A: well-differentiated HCC, B: moderately-differentiated HCC, C: poorly-differentiated HCC, Rim-enhancement AP: rim-enhancement in arterial phase

### Texture analysis and tissue classification

As showed in Tables 4, 262 texture features derived from T2, HBP, AP and EP images were obtained and categorized into histogram (*n* = 10), GLCOM (*n* = 220), GLRLM (*n* = 20) and wavelet transform (*n* = 12). The frequency of each feature category of T2-weighted images and each phase of Gd-EOB-DTPA enhanced images extracted by FPM was showed among poorly-differentiated, well-differentiated and moderately-differentiated HCC. The GLCOM-based texture features were most frequently extracted with three phases for poorly- verse well-differentiated HCC, poorly- verse moderately-differentiated HCC and well- verse moderately-differentiated HCC.

**Table 4:** The frequency of each feature category extracted by FPM from AP, EP, HBP and T2 images among poorly-differentiated, well-differentiated and moderately-differentiated HCC

Texture features	A verse C				B verse C				A verse B			
	AP	EP	HBP	T2	AP	EP	HBP	T2	AP	EP	HBP	T2
Histogram (n = 10)	4	5	2	5	4	2	1	5	3	1	1	0
GLCOM (n = 220)	15	10	17	142	14	18	19	45	16	16	19	40
GLRLM (n = 20)	5	9	6	13	5	4	3	4	6	6	5	7
Wavelet transform (n = 12)	6	6	5	9	7	6	7	3	5	7	5	8

Note: A: well-differentiated HCC, B: moderately-differentiated HCC, C: poorly-differentiated HCC;

*AP* arterial phase, *EP*: equilibrium phase images, and *HBP* hepatobiliary phase

The tissue classification results were demonstrated across the T2, AP, EP and HBP in Table 5. The misclassification rate of NDA was excellent for each phase of the three groups, with the misclassification rate ranging from 3.33 to 14.93%. The misclassification rate of LDA was rank secondly to NDA, with the classification rate range from 4.92 to 33.75%. Both of the misclassification results of RDA and PCA were fair or poor.

**Table 5:** Misclassification rate of texture analyses from AP, EP, HBP and T2 images among poorly-differentiated, well-differentiated and moderately-differentiated HCC

	A verse C				B verse C				A verse B			
	AP	EP	HBP	T2	AP	EP	HBP	T2	AP	EP	HBP	T2
RDA (%)	44.26	34.43	50.82	48.33	55.00	50.50	47.50	46.25	34.33	46.27	44.78	47.76
PCA (%)	42.62	36.07	47.57	50.00	53.75	53.75	48.75	40.00	28.36	37.31	44.78	40.30
LDA (%)	14.75	4.92	9.84	10.00	17.50	33.75	33.75	20.00	26.87	23.88	11.94	26.87
NDA (%)	11.48	4.92	6.56	3.33	10.00	13.75	13.75	12.50	8.96	14.93	4.48	7.46

Note: *RDA* raw data analysis, *PCA* principal component analysis, *LDA* linear discriminant analysis, *NDA* nonlinear discriminant analysis

A: well-differentiated HCC, B: moderately-differentiated HCC, C: poorly-differentiated HCC; *AP* arterial phase, *EP* equilibrium phase images, and *HBP* hepatobiliary phase

### ROC-analysis

**Fig. 2:**
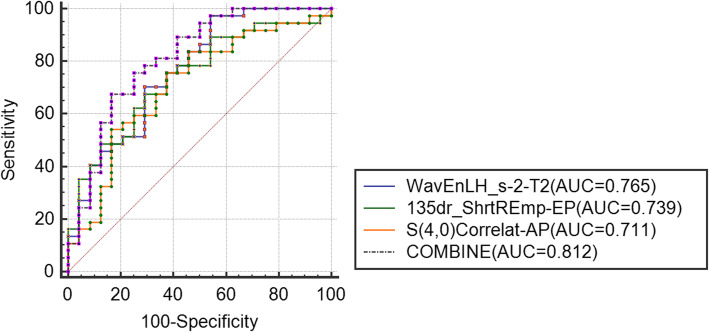
ROC curves for differentiating the poorly- and well-differentiated HCC. The ROC curves were drawn according to the texture features with the highest AUC derived from T2, EP and AP. And the ROC curve of the combined texture features was shown as COMBINE

**Fig. 3:**
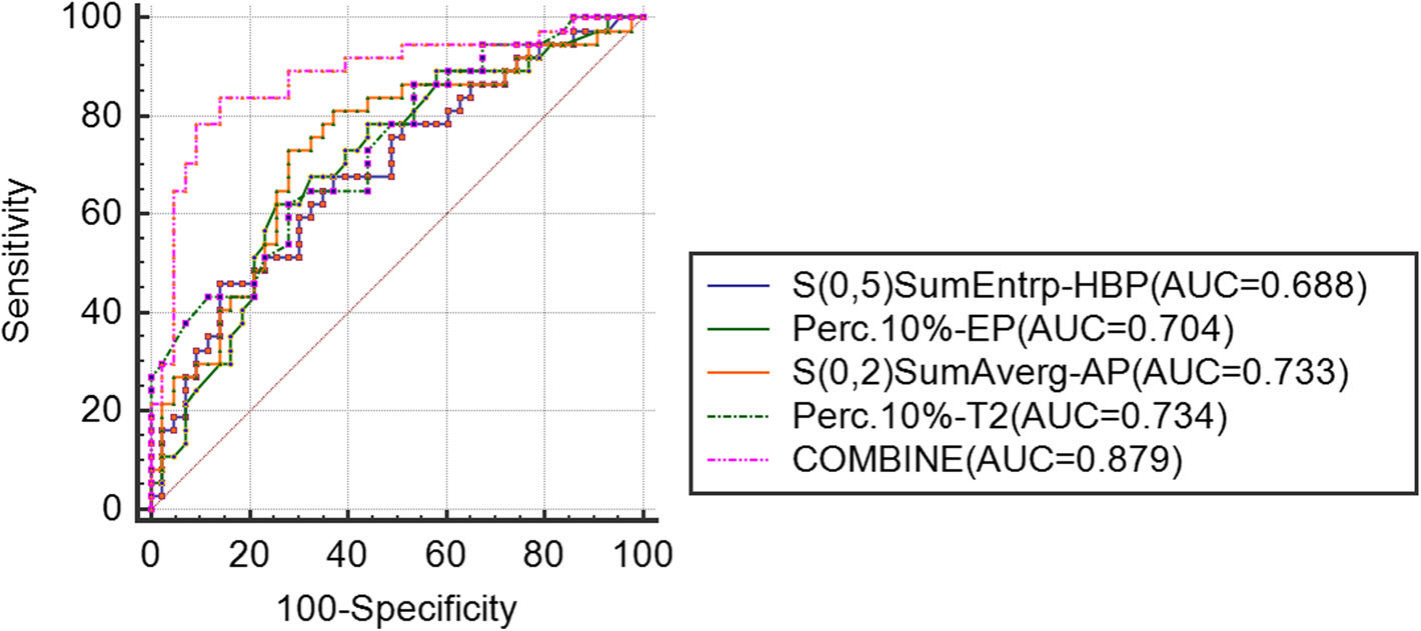
ROC curves for differentiating the poorly- and moderately-differentiated HCC. The ROC curves were drawn according to the texture features with the highest AUC derived from T2, AP, EP and HBP. And the ROC curve of the combined texture features was shown as COMBINE

**Fig. 4:**
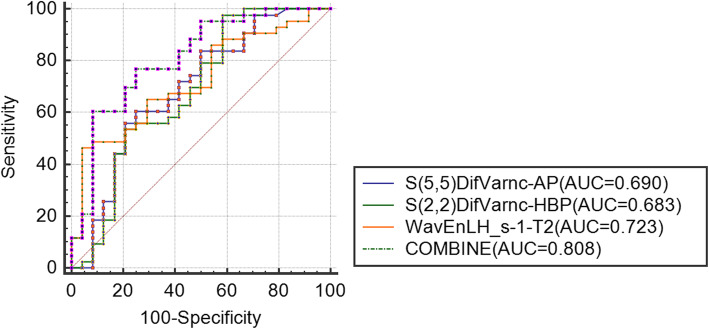
ROC curves for differentiating the well- and moderately-differentiated HCC. The ROC curves were drawn according to the texture features with the highest AUC derived from T2, AP and HBP. And the ROC curve of the combined texture features was shown as COMBINE

The AUC of each texture feature was calculated. The ROC curves of the best combined diagnoses were demonstrated in Figs. 2, 3 and 4. As showed in Fig. 2, the combine AUC value (combining texture features from T2, AP and EP) was 0.812, higher than that of any single texture feature from each phase, to differentiate poorly- from well-differentiated HCC (accuracy = 0.77). As showed in Fig. 3, the combine AUC value was 0.879 (accuracy = 0.85), to differentiate poorly- from moderately-differentiated HCC, and as showed in Fig. 4, the combined AUC value was 0.808 (accuracy = 0.746) to differentiate moderately- from well-differentiated HCC.

The ROC analyses of combined tumor size and texture features were demonstrated in Table 6. “COMBINE” presented the combination of texture features derived from different phases. As showed in the Table 6, the AUC of tumor size was the lowest and the COMBINE AUC value was the highest. With combining tumor size and texture features, the COMBINE AUC values were the same as those without combining tumor size, in poorly- verse moderately-differentiated HCC and poorly- verse well-differentiated HCC, while the COMBINE AUC value was increased from 0.808 to 0.833 in moderately- with well-differentiated HCC (*p* = 0.314).

**Table 6:** The AUC of the texture features and tumor size among poorly-differentiated, well-differentiated and moderately-differentiated HCC

A verse C			B verse C			A verse B		
Texture features	AUC	accuracy	Texture features	AUC	accuracy	Texture features	AUC	accuracy
S(4,0)Correlat-AP	0.711	0.656	S(0,2)SumAverg-AP	0.733	0.700	S(5,5)DifVarnc-AP	0.690	0.642
135dr_ShrtREmp-EP	0.739	0.721	Perc.10%-EP	0.704	0.688	S(2,2)DifVarnc-HBP	0.683	0.731
WavEnLH_s-2-T2	0.765	0.705	Perc.10%-T2	0.734	0.663	WavEnLH_s-1-T2	0.723	0.657
COMBINE	0.812	0.770	S(0,5)SumEntrp-HBP	0.688	0.638	COMBINE	0.808	0.746
Tumor Size	0.649	0.639	COMBINE	0.879	0.850	Tumor Size	0.517	0.642
COMBINE+Tumor Size	0.812	0.770	Tumor Size	0.660	0.600	COMBINE+Tumor Size	0.833	0.791
			COMBINE+Tumor Size	0.879	0.825			

Note: A: well-differentiated HCC, B: moderately-differentiated HCC, C: poorly-differentiated HCC; *AP* arterial phase, *EP* equilibrium phase images, and *HBP* hepatobiliary phase. COMBINE: demonstrates the AUC of the combination of statistically significant texture features derived from T2 weighted imaging and different phases of Gd-EOB-DTPA-enhanced MRI

### Correlation between texture features and differentiated degree of HCC

Perc.10% was positively correlated with the differentiated degree of HCC in AP (*r* = 0.276, *p* = 0.005), while 135dr_ShrtREmp was negatively correlated with the differentiated degree of HCC in EP phase (*r* = − 0.305, *p* = 0.002) and S(3,0) SumEntrp was negatively correlated with the differentiated degree of HCC in T2 phase (*r* = − 0.306, *p* = 0.02).

## Discussions

As previous studies showed, the diameter of HCC was an important factor to predict the pathological grade of HCC. Lee et al. [22] and Martins et al. [23] suggested that the diameter of most moderately-differentiated HCC was larger than well-differentiated HCC. Our present study found that the diameter of poorly-differentiated HCC was larger than that of moderately-differentiated and well-differentiated HCC. However, there was no significant difference of diameter between poorly and well-differentiated HCC in present study, which was not in consistence with the Martins’. It may be due to the heterogeneity of the tumor cells and the individual differences of tumor growing patterns, as well as the limited sample size. Additionally, it was found that the diagnostic efficiency of tumor size was lower than those of the texture features in present study, which was consistent with previous study [24], suggesting the critical role of texture analysis in identifying the differentiated degree of HCC.

The differential degree of HCC was the most important factor that affect the prognosis of the patients. In this study, the patients were grouped into poorly, moderately and well-differentiated group based on the histopathological outcomes, and whether the texture features could successfully differentiate the subtypes of HCC were explored. Texture analysis was a method that could quantize the information provided by the images. Some studies verified that texture analysis had the potential to identify the histopathological type of neoplasm, such as the breast cancer and renal tumor [21, 25]. However, there were no studies to explore the value of texture features derived from multi-phase of Gd-EOB-DTPA-enhanced MRI and T2WI in predicting the histopathological grades of HCC yet.

In recent years, researchers gradually realized that the substantial quantitative features were increasingly important in the tumor diagnoses, not merely the application of qualitative features such as margin, signal intensity, capsule of the tumor and so on [26]. Mazda was a software package which provided a complete path for quantitative analysis of image texture. It included image analysis, texture features extraction, data classification, analysis automation and other functions [20]. Substantial information obtained by Mazda, might differentiate the pathological grade of tumor. Previous study analyzed the texture features to predict the OS of the patients with advanced HCC [27]. Our study attempted to identify the histopathological grade by texture analysis.

B11 module provided four procedures, RDA, PCA, LDA and NDA, to analyze the selected thirty features. In present study, the classification rate of NDA was excellent. It suggested that texture analysis was a reliable method to identify the poorly-, moderately- and well-differentiated HCC. Although LDA was recommended as an optical method, NDA was more excellent than LDA in present study, which was in consistent with Li Y’s study [28]. This might be due to the non-linearity of the clinical data which was obtained in a random way. And the inconformity of the misclassification rate from the texture analysis of different image sequences, might result from the different histological components and enhancement patterns among the subtypes of HCC [21].

The GLCOM-based features which described the spatial dependence of gray value in image were most frequently extracted than other texture features of other categories regardless of the phase of MRI and groups [28, 29]. It was implied that the different pathological grades might impact the gray value of the image. Additionally, the tremendous number of texture features included in the GLCOM (*n* = 220) might lead to the high frequency of the extracted text features [21]. The GLRLM was secondly selected by texture analysis, which demonstrated the pixel runs with the same grey level values in a given direction and depicted intensity homogeneity in a given direction [28]. The result might suggest that the intensity homogeneity between poorly-, moderately- and well-differentiated HCC was different. The GLCOM-based features generated from AP was noticeably different between groups.

In present study, it was found that histogram-derived parameter —— Perc.10% of AP was positively correlated with the differentiated degree of HCC. It was suggested that the signal intensity in AP imaging was detectably higher with a higher differentiated degree. However, as previous study showed, HCC with a higher differentiated degree was prone to have lower arterial supply. The individual differences of HCC arterial supply might lead to this discrepancy [30]. 135dr_ShrtREmp was a GLRLM-based texture feature to measure the heterogeneity and SumEntrp was a parameter to measure randomness and heterogeneity of the studied region. 135dr_ShrtREmp of EP and SumEntrp of T2 were negatively correlated with differentiated degree of HCC, suggesting that the poorly-differentiated HCC was most heterogeneous among different differentiated grades of HCC both in EP and T2 phase [25, 31]. However, there was no statistical difference of signal (a routine MR feature) in different differentiated degrees of HCC as showed in Table 3. Therefore, the texture analysis was supposed to be a preciser method to evaluate the differentiated degree of HCC than traditional MRI imaging characteristics.

As showed in Table 6, the COMBINE (combined S(0,2) SumAverg of AP, Perc.10% of T2, Perc.10%-EP and S(0,5)SumEntrp-HBP) AUC value was the highest when moderately- verse poorly-differentiated HCC. S(0,2) SumAverg and Perc.10% reflected the signal intensity of the lesion, and the S(0,5) SumEntrp reflected randomness and heterogeneity of the studied region. Therefore, the signal intensity of T2, AP and EP and the heterogeneity of HBP were supposed to be important to predict the differentiated degree of HCC. The COMBINE (combined S(4,0)Correlat-AP, 135dr_ShrtREmp-EP and WavEnLH_s-2-T2) AUC value was the highest when well- verse poorly-differentiated HCC, while the COMBINE (combined S(5,5)DifVarnc-AP, S(2,2)DifVarnc-HBP and WavEnLH_s-1-T2) AUC value was the highest when well- verse moderately-differentiated HCC. All the above features reflected the heterogeneity of lesion. Both the signal intensity and heterogeneity of HCC valued in identifying the differentiated degree of HCC. In addition, the AUC of tumor size was the lowest, suggesting that the texture features analysis was preciser than tumor size in identifying the differentiated degree of HCC. These results suggested that radiologists should focus on the signal intensity and heterogeneity of lesion in clinical diagnosis.

GPC-3 was a member of the glypican family, which influenced cell growth, differentiation, and migration [32]. Previous studies demonstrated that higher GPC-3 expression level in HCC was a risk factor for shorter overall survival and GPC-3 expression level in poorly-differentiated tumor cells was higher than that in moderately- and well- differentiated HCC [32–34]. But there was no significant difference of the expression of GPC-3 among poorly-, moderately- and well- differentiated HCC in present study. The small sample size was supposed to be the reason of this discrepancy.

There were some limitations in our study. Although we adopted strict inclusion and exclusion criteria in this retrospective study, selection bias was still inevitably. Second, the sample size was relatively small which need to be enlarged in the future study. Third, the ROI (tumor contour) was manually delineated on the slice containing the maximum diameter, which led to the lack of three-dimentional information of the tumor.

## Conclusions

In conclusion, the texture analysis of multiphase Gd-EOB-DTPA-enhanced MRI and T2WI were noninvasive and reliable quantitative technique to predict the differentiated grade of HCC. Texture analysis performed better than the tumor size in discriminating the differentiated grade of HCC. The signal intensity and heterogeneity of HCC were valued in identifying the differentiated degree of HCC.

## Data Availability

The datasets analyzed during the current study are available from the corresponding author on reasonable request.

## Abbreviations

HCC: Hepatocellular carcinoma
GPC-3: Glypican-3
AFP: Alpha fetoprotein
ALT: Alamine aminotransferase
AST: Aspartate transaminase
T2WI: T2 weighted imaging
ROC: Receiver operating characteristic
AUC: Area under the curve
TBIL: Total bilirubin
AP: Hepatic arterial phase
PVP: Portal venous phase
EP: Equilibrium phase
HBP: Hepatobiliary phase
ROI: Region of interest
GLCOM: The grey-level co-occurrence matrix
GLRLM: The grey-level run-length matrix
PCA: Principal component analysis
LDA: Linear discriminant analysis
NDA: Nonlinear discriminant analysis
RDA: Raw data analysis
